# Mr. Weeden Cooke, on the Treatment of Gonorrhœa and Gleet without Capabia

**Published:** 1860-07

**Authors:** 


From the Transactions of the Harveian Society.

### MR. WEEDEN COOKE, ON THE TREATMENT OF GONORRHŒA AND GLEET WITHOUT COPAIBA.

After referring to Sir Astley Cooper's method of treating gonorrhœa, the author stated his belief that he was only giving utterance to a largely-acknowledged experience when he ventured to affirm that copaiba, in the treatment of gonorrhœa was not only unnecessary, but that it was, in a great many instances, injurious, and that in all it was offensive to the last degree. During the last fifteen years, at least 6000 cases of gonorrhœa had come under the author's care at the Royal Free Hospital, and he had availed himself of these large opportunities to test all the methods of treatment which had been suggested, in order to arrive at the safest, quickest, most efficacious, and least disagreeable mode of curing the disease. As introductory to the subject of treatment, Mr. Cooke discussed the subject of the cause of chordee, the reason for the scalding of the urine, the distinction between the true and spurious gonorrhœa, and the time when infection may or may not be apprehended. The conclusions he arrived at were—

1st. That chordee, in ninety-nine cases out of a hundred, was due to spasm, and not to effusion of lymph; and that cubebs, acting as an antispasmodic formed the most efficacious remedy for this symptom.

2ndly. That the scalding was the result of the acid urine passing over the highly inflamed mucous surface of the urethra; and that this was to be remedied by the administration of the alkaline carbonates for the purpose of neutralizing the acidity of the urine, thus removing the principal cause of the continuance of the inflammation.

3d. That in all disputed cases, the true gonorrhœa may be known from the spurious by the presence of redness, heat, pain, and swelling, together with a purulent discharge, more or less green and offensive; whilst a discharge produced by connection with a person who has leucorrhœa, or has recently been confined, will not be accompanied by these inflammatory signs, and the discharge will be milky in consistence and color, differing much from the thick, purulent discharge of the veritable gonorrhœa.

4th. The time when infection may or may not be apprehended, was discussed in its incubative stage, and at its close. A case was given, showing that the disease may be caught from one person, and not communicated to another two nights after, because the purulent discharge had not commenced at the time of second intercourse. Respecting infection at the close of the disease, the author had been enabled, from experience, to establish, as a law for his own guidance, that gleet; i. e., a mucous discharge from the urethra, consequent on gonorrhœa—does not set up gonorrhœa in another person; but that, whilst any pus is to be found in the discharge, there is probability of infection.

Passing on to the subject of treatment, Mr. Cooke said that, upon inquiry at the London Custom House, he found that 118,396 pounds of copaiba were admitted into the port of

London only, during the first ten months of the year 1859. If this be administered at the rate of half a drachm three times a day, and supposing each patient takes it for three weeks, we have here copaiba enough to treat 473,584, or close upon half a million persons, and that in ten months, only, of the year. Considering how often it fails to cure the disease; how frequently it is rejected by, or, at any rate, disorders the stomach; how tell-tale and disagreeable is its odor from the mouth and skin; how occasionally it produces a papular eruption all over the body; and that, in many instances, swelled testicle and stricture may be traced to its irritating influence, while gonorrhœa, rheumatism, and ophthalmia, have been attributed to its administration: "considering all these objections," the author remarked, "is it not extraordinary that this disgusting medicine continues so long to hold its ground?" The abortive treatment by strong injections of nitrate of silver had proved a failure, because in some instances inflammation of the bladder had resulted. The treatment by diluents was slow in its action, and not readily employed by persons engaged in active business. That by diuretics was scarcely more successful, whilst the administration of saline aperients was generally attended with an aggravation of the ardor urinæ as well as chordee. The treatment which had been most successful in the author's experience was the chemical treatment by the alkaline carbonates, given with a view of neutralizing the acid in the urine. Thus one great source of irritation was removed from the inflamed urethra, and the subsidence of the inflammation, which nature would effect, was allowed to take place. As auxilliaries, especially where there is œdema of the prepuce, lead lotions, and elevation of the penis against the abdomen, were commended. The inflammation having subsided, and a muco-purulent discharge being left, the author had found, after giving trial to all the injections which have been at any time in vogue, that the chloride of zinc, introduced into this branch of practice by Mr. Lloyd, of St. Bartholomew's, was the most efficacious of any in curing the disease, and that with less discomfort and in a much shorter time than by any other means. Since employing this treatment, he had had little, if any, orchitis amongst his patients. The strength of the injection he most commonly employed, was two grains to the ounce, but in some instances one grain to the ounce was sufficient. Whilst advocating this treatment in persons of healthy constitution, it was necessary to completely change it in others. In the strumous, in the dyspeptic, in those of dissipated habits, and where the diseased person is an old offender, the alkaline carbonates are not called for, because either the urine is not acid or the inflammation does not run high. In such cases the tincture of iron or sulphuric acid and bark, or gentian, or calumba, may be advantageously employed from the commencement; and the chloride-of-zinc injection in these cases is also of the utmost value in rapidly overcoming the disease. Respecting diet, the author considered that after the subsidence of the inflammatory symptoms, scarcely any restriction need be enforced, and that beer or wine in moderate quantities may be advantageously used by those who are accustomed to these beverages. He had found long-established cases of gleet yield readily to the chloride-of-zinc injection, accompanied with tonic treatment and generous living.

In conclusion he would rejoice if the treatment he had found so serviceable should be followed out by others, and thus assist in banishing altogether from surgical practice the use of so nauseous a drug as cobaiba.—*London Lancet.*

